# Feel Safe and Money is Less Important! Hypnotic Suggestions of Safety Decrease Brain Responses to Monetary Rewards in a Risk Game

**DOI:** 10.1093/texcom/tgaa050

**Published:** 2020-08-20

**Authors:** Barbara Schmidt, Elisa Hoffmann, Björn Rasch

**Affiliations:** Institute of Psychology, University of Jena, 07743 Jena, Germany; Institute of Psychology, University of Jena, 07743 Jena, Germany; Department of Psychology, University of Fribourg, CH-1701 Fribourg, Switzerland

**Keywords:** ERP, hypnosis, monetary rewards, P300, risk, safety, suggestion

## Abstract

When a stimulus is important, the corresponding brain responses increase, especially the P300 brain response. This is true for all kinds of important stimuli, also monetary rewards. In our study, we developed a hypnotic suggestion to reduce the subjective importance of monetary rewards. As successful suggestions do not contain negations, we suggested participants to feel safe during hypnosis instead of suggesting that money is not important anymore. We predicted lower P300 amplitudes when participants feel safe during hypnosis. We tested 24 highly suggestible participants playing a risk game in 2 conditions with monetary rewards while we measured their EEG brain responses. In the safety condition, we induced a hypnotic state and suggested that participants feel safe. In the control condition, participants played the risk game without hypnosis. Here we show that participants felt significantly safer in the safety condition and showed significantly lower P300 amplitudes to monetary rewards. Risk behavior did not differ significantly between conditions. Our results are important for substance use disorders, as decreased P300 responses to substance-related stimuli are associated with less craving and better abstinence. Therefore, we conclude that suggestions to feel safe during hypnosis might work as a treatment for individuals with substance use disorders.

## Introduction

Our brains have developed efficient ways to guide our motivation and attention towards important stimuli. When a stimulus is important, brain responses to this stimulus are enlarged, also P300 amplitudes in the EEG event-related potential. There are many factors that make stimuli important. When we are hungry, it is important to find food, so food stimuli get very important and elicit higher P300 amplitudes (Nijs et al. 2008, 2010; Stockburger et al. 2009). When we like smoking, smoking-related stimuli are important for us and elicit higher P300 amplitudes (Littel et al. 2012). A stimulus that is rewarding for most of us is money. Receiving money as a reward increases motivation and attention, both inside and outside the laboratory (Jenkins et al. 1998; Small et al. 2005). In an easy stimulus response task, the amplitude of the P300 after a stimulus that promised a monetary reward for a correct button press was significantly increased compared to a control stimulus (Begleiter et al. 1983). As the amplitude of the P300 is increased for stimuli with higher incentive value, it can be assumed that it reflects participants’ subjective motivation (Begleiter et al. 1983).

Using monetary rewards as incentives in research paradigms has many advantages. Money is easy to quantify and easy to deliver. Monetary rewards are also often used in neuroeconomic paradigms. In our lab, we developed a risk game where participants make risky decisions to get monetary rewards (Schmidt et al. 2013; Schmidt and Hewig 2015; Schmidt, Mussel et al. 2017; Schmidt et al. 2018; Schmidt, Kessler, Hecht et al. 2019; Schmidt, Kessler et al. 2019). In each trial of the risk game, participants choose a riskier or less risky option which are equal concerning expected values and then receive a higher or lower monetary reward. To get the highest possible monetary reward, participants must choose the riskiest option that can also result in receiving no money at all. Higher monetary rewards elicit higher P300 amplitudes in the risk game (Schmidt, Hecht et al. 2017). In the present study, we use monetary rewards as incentives that reliably elicit participants’ motivation and attention and develop an intervention that reduces the attraction of monetary rewards, which is measurable in decreased P300 amplitudes.

To reduce the importance of monetary rewards, we need to change participants’ general motivational state. Feeling safe is associated with a very satisfied motivational state (Maslow 1943) that has the potential to reduce the importance of monetary rewards. This reduction in subjective importance should be reflected in reduced P300 responses to monetary rewards. Please note that the direct suggestion “money is not important anymore” will very likely have paradoxical effects, as shown in the famous study by Wegner et al. (1987). Participants who were told “Don’t think about a white bear” thought more about a white bear than participants who were told to think about a white bear. That is why we used the suggestion to be at a safe place and predicted that the feeling of safety will make monetary rewards less relevant, leading to reduced P300 amplitudes.

To induce a feeling of safety, we developed an intervention that uses hypnosis and the suggestion of a safe place. Suggesting feeling safe is a very effective and common method in hypnotherapy (Arntz 2011; Schmidt et al. 2020). Suggestions work better when participants are hypnotized (Hilgard and Tart, 1966), and positive suggestions during hypnosis can reduce anxiety and stress in medical contexts (Tefikow et al. 2013; Kekecs and Varga 2014; Schmidt et al. 2020). Therefore, we combined hypnosis with the suggestion of being at a safe place to achieve the best results.

Feeling safe should only affect the importance of monetary rewards, not the perception of reward magnitude. That means, we expect that participants will still differentiate between lower and higher rewards while all rewards are less important to them. This is in line with neuronal dissociation theories of hypnosis (e.g., Kirsch and Lynn 1998) and previous results from our laboratory (e.g., Schmidt, Hecht et al. 2017). In our previous study, we suggested participants that they see a wooden board in front of their eyes blocking their vision on a screen. ERP components reflecting early visual processing stages were not affected by this suggestion, while P300 amplitudes reflecting later visual processing stages were significantly reduced (Schmidt, Hecht et al. 2017). In line with these results, we predicted that ERP components reflecting earlier processing stages of monetary rewards will not be affected by the suggestion of safety, while P300 amplitudes will be significantly reduced. We measure earlier processing stages of monetary rewards via the feedback-related negativity (FRN; Miltner et al. 1997). The FRN differentiates between lower and higher monetary rewards, showing more negative amplitudes for lower monetary rewards (Schmidt, Mussel et al. 2017; Schmidt, Kessler, Hecht et al. 2019). The neuronal dissociation will be revealed by a non-significant condition effect for FRN amplitudes and a significant condition effect for P300 amplitudes.

Feeling safe might also have an effect on risk behavior in our risk game. When participants are anxious, they make less risky decisions in this risk game (Schmidt et al. 2018; Wake, Wormwood and Satpute 2020). On the other hand, participants who wear a bike helmet that can be interpreted as an indirect safety prime show risk indifference in their decision-making behavior (Schmidt, Kessler et al. 2019). Based on these findings, we expected that feeling safe should lead to riskier decisions in the risk game.

Taken together, the aim of this study is to test if an induced feeling of safety affects risk behavior and reduces P300 brain responses to monetary rewards in a risk game. After the suggestion of safety, participants played a risk game with monetary rewards while we recorded their EEG. Participants played the same risk game also in a control condition without hypnosis and the suggestion of safety. We expected riskier behavior and lower P300 amplitudes after monetary rewards in the safety condition compared to the control condition. Reduced P300 responses to monetary rewards are associated with less attention and motivation, indicating lower subjective importance of those stimuli. This in turn is important for substance use disorders, where substance-related stimuli evoke high P300 amplitudes associated with high subjective importance. When feeling safe makes participants care less about monetary rewards, this effect could be used to reduce the subjective importance of substance-related stimuli in substance-dependent individuals.

## Materials and Methods

### Participants

In a previous hypnosis study conducted in our laboratory, the within-subjects effect size for the reduction of the P300 amplitude in a visual oddball task was *d* = 0.7 (Schmidt, Hecht et al. 2017). With a power level of 0.95 and an alpha level of 0.05, 24 participants are required to detect an effect according to G*power (Faul et al. 2007). Therefore, we collected data from 24 participants (12 female) whose mean age was 25.2 years (range 19–40 years). We selected these participants from a pool of 122 participants who were pre-tested in a separate experimental session for their level of hypnotic suggestibility using the Harvard Group Scale of Hypnotic Susceptibility, Form A (HGSHS:A; Shor and Orne 1963). In the HGSHS:A, the experimenter hypnotizes a group of participants and then presents 12 suggestions. The induction part of the HGSHS:A contains suggestions to close the eyes, to focus on one’s breathing and to relax. The 12 items of the HGSHS:A include ideomotor suggestions like hand lowering, challenge suggestions like arm immobilization, and cognitive-delusory suggestions like amnesia (McConkey et al. 1980; Piesbergen and Peter 2006). In our study, Barbara Schmidt administered the HGSHS:A in life sessions with 8–10 participants. She also induced hypnosis and provided the safety suggestions in the EEG session later. Like this, a positive relationship between hypnotist and participants was promoted which further improves the effect of hypnosis and suggestions (Gfeller, Lynn and Pribble 1987). Dependent on the number of suggestions participants successfully complete in the HGSHS:A, they are assigned a score of 0 to 12. Internal consistency of the HGSHS:A was Cronbach’s alpha = 0.69 in our sample. For our study, we invited participants with hypnotic suggestibility scores of at least 8 out of 12 (M = 8.6, range 8–11), indicating high hypnotic suggestibility. Participants were paid according to the outcomes in the risk game and another game that will be described elsewhere. The average payment was 23.5 Euro (SD = 0.2 Euro). The study was carried out in accordance with the Declaration of Helsinki and was approved by the ethics committee of the Friedrich Schiller University of Jena.

### Apparatus

The experimental tasks were programmed and presented in Presentation® software (Neurobehavioral Systems, Inc., Berkeley, CA, www.neurobs.com). Statistical analyses were computed with R (R Development Core Team 2020). To compute within-subject effect sizes, we used Cohen’s *d* according to the formula provided by Lakens (2013) in equation 7. For ANOVA within-subject effect sizes, we used generalized eta squared as recommended by Bakeman (2005).

### Procedure

**Figure 1:**
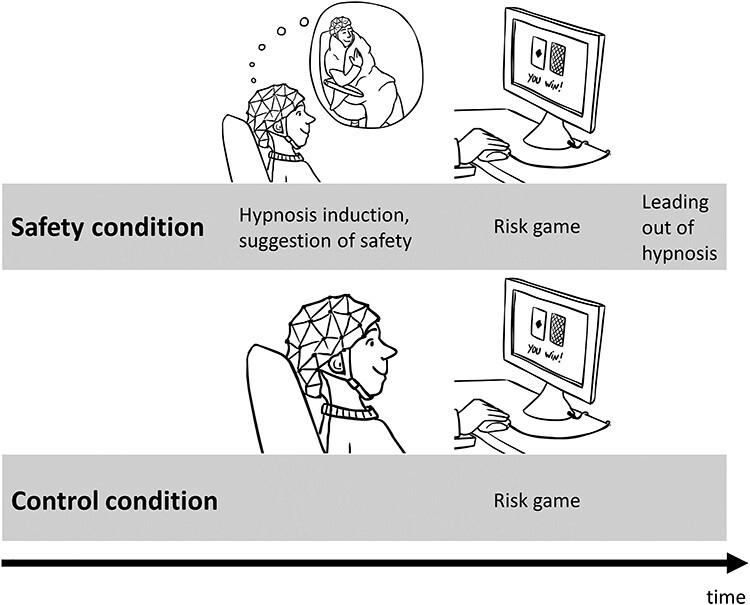
Illustration of the study design with the safety condition and the control condition.

Participants read a participant information sheet including the description of the risk game and provided informed consent in the beginning of the experiment. The experimenter showed a paper version of the playing cards that would occur in the game later and explained their meaning once again. Then, an electrode cap with 64 electrodes (EASYCAP, Woerthsee-Etterschlag, Germany) for recording the EEG was placed on the participants’ head. Participants were seated in a dimly lit room on a comfortable chair, approximately 100 cm in front of a computer monitor. Participants played the risk game first and then another game that will be reported elsewhere, in both a safety condition and a control condition, the order of which was counter-balanced across participants, see Figure 1. In the safety condition, the experimenter conducted a hypnosis induction according to the Stanford Hypnotic Susceptibility Scale, Form C (Weitzenhoffer and Hilgard 1962). During the hypnosis induction, which lasted about 20 minutes, participants were instructed to close their eyes, relax and breathe deeply. The experimenter then administered the first item of the Stanford Hypnotic Susceptibility Scale, Form C (Weitzenhoffer and Hilgard 1962) to see if participants responded to this suggestion. This item entails that the participants stretch out their right arm. Then, the experimenter suggests that the arm gets heavy as if participants carry a heavy weight in their hand. When the hand moved downwards at least 15 cm, the item was scored as passed. Then, the experimenter suggested safety via the imagination of a safe place. She told participants that she takes them for a journey to a place where they feel completely safe. We provide audio and text files of the safety suggestion in German and English as Supplementary Material. Here we present a few sample sentences of the safety suggestion: “Allow yourself to feel completely safe. You can let yourself go completely. Think again of the place in your body where you feel the feeling of safety most clearly. From there it radiates out into all parts of your body like sunbeams. And you know how strong the sun can shine. It is a thoroughly pleasant feeling. You are completely filled with it. The feeling gets stronger and stronger. It becomes so big that you can feel it even outside your body like a safe shell. The feeling of safety is like a blanket that makes you feel safe and secure. You are now completely enveloped by the feeling of safety.” Figure 1 illustrates the feeling of safety which was also described as the warm feeling of being cuddled into a blanket.

After the suggestion of safety, the experimenter asked participants to open their eyes. Then, the participants played the risk game and another game as described below. Between both games, the suggestion of safety was repeated to re-intensify the feeling. Each game lasted about 10 minutes. After completing the games, the participants were led out of the hypnotic state. The experimenter asked the participants how strongly they experienced the feeling of safety. The scale ranged from 1 for “not at all” to 5 for “I felt very safe”. After answering this question, participants filled in the German version of the Inventory Scale of Hypnotic Depth (ISHD-D; Riegel et al. 2018; original English version by Field 1965). The ISHD-D contains 36 items that are to be scored from 1 for “not at all true” to 4 for “completely true”. One sample item is “Time stood still”, another item is “Everything happened automatically”. We obtained a Cronbach’s alpha of 0.86 in our sample, replicating findings of Riegel et al. (2018).

In the control condition, participants also played both games. The control condition lasted about 30 minutes altogether, while the safety condition lasted about 60 minutes, as participants were hypnotized and suggested to be at a safe place before and filled in the ISHD after playing the games. After completing the EEG study, participants washed their hair and signed the payment form, so the whole EEG session lasted about 2 hours.

*Risk game.* Participants played the same risk game as described in earlier studies of our group (Schmidt et al. 2013; Schmidt and Hewig 2015; Schmidt, Mussel et al. 2017; Schmidt et al. 2018; Schmidt, Kessler, Hecht et al. 2019; Schmidt, Kessler et al. 2019). Participants played 60 trials of the risk game in each condition. After the risk game, participants rated all response options (11 or 0 cents, 10 or 1 cents, 9 or 2 cents, 8 or 3 cents, 7 or 4 cents, 6 or 5 cents) according to their perceived valence, arousal and riskiness. Valence and arousal were measured using the Self-Assessment Manikin (Bradley and Lang, 1994). The rating scales ranged from 1 to 9 with higher scores indicating more positive, more arousing and riskier evaluations, respectively.

**Figure 2:**
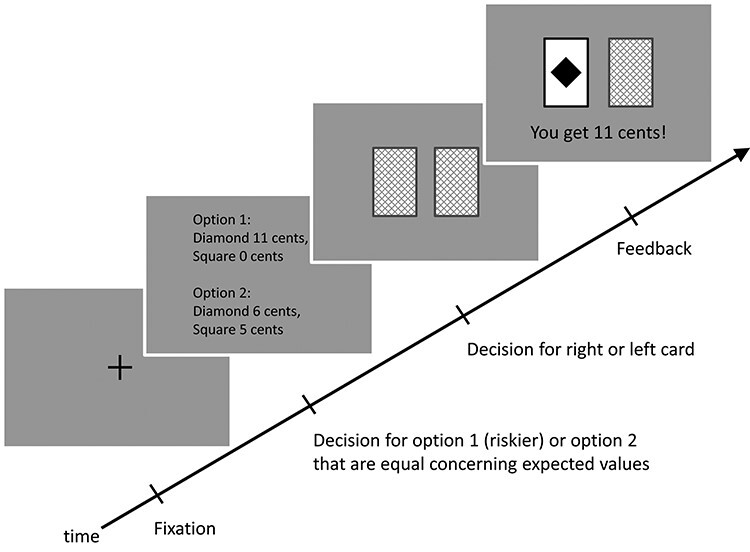
Time course of one trial in the risk game.

At the beginning of each trial, a fixation cross was shown for a random interval of 1000 to 2000 ms (Fig. 2). Then, 2 options were presented. One option was riskier, the other option less risky. Both options consisted of 2 monetary rewards. The expected value of both options was always 5.5 cents, and the degree of riskiness differed between the options, from 11 cents versus 0 cents as the riskiest option and 6 cents versus 5 cents as the safest option. Participants always chose between the riskiest option (11 cents vs. 0 cents) and one of the other options (10 or 1 cent, 9 or 2 cents, 8 or 3 cents, 7 or 4 cents, 6 or 5 cents). All option pairs were presented in random order and at random locations left or right on the monitor screen. Participants were required to choose an option by pressing one of 2 buttons. After another random interval of 500 to 1000 ms, 2 cards were shown face-down (Fig. 2). Then participants had to choose one of the cards by pressing one of 2 buttons with their right hand. After another random interval of 500 to 1000 ms, the back of the selected card was shown, displaying either a diamond that indicated the higher monetary reward (positive feedback) or a square indicating the lower monetary reward (negative feedback), together with the statement “You get XX cents!” for 1500 ms. Unbeknownst to the participants, on 50% of the trials the monetary feedback was positive and on the other 50% the monetary feedback was negative, delivered at random independently of their choices. All stimuli in the risk game occupied about 10° of visual angle horizontally and 5° vertically. At the end of the game, the total accumulated reward was presented to the participants. Participants were paid the corresponding amount.

### E‌EG Recording and ERP Quantification

The EEG was recorded from 64 Ag/AgCl scalp electrodes, using 2 BrainAmp DC amplifiers (Brain Products GmbH, Gilching, Germany). Impedances were below 10 kΩ and electrode recordings were referenced to the electrode FCz online. The data were band-pass filtered during recording from 0.016 Hz to 250 Hz and sampled at 500 Hz. For offline data processing, EEGLAB (Delorme, and Makeig 2004) running under the MATLAB environment (The MathWorks, Inc.) was used.

EEG artifacts were corrected using independent component analysis (ICA) as proposed by Debener et al. (2010). We removed eye-related artifact components by back-projection of all remaining components. The artifact-corrected data were then re-referenced to the mean of the voltages recorded at electrode locations TP9 and TP10. For ERP analysis, the data were low-pass filtered with 20 Hz, segmented into epochs from −200 ms to 800 ms around reward feedback onset, and baseline-corrected (−200 ms to 0 ms). Epochs with residual artifacts were removed. We performed a statistical artifact correction rejecting all epochs deviating more than 4 standard deviations from all epochs of the specific participant and event. The artifact rejection criteria were joint probability and kurtosis using the algorithm pop_jointprob() and pop_rejkurt() implemented in EEGLAB version 13.6.5b. The mean number of artifact-free epochs was 27.3 (SD = 1.4) and the number of artifact-free trials did not differ between conditions or monetary reward feedback as revealed by an analysis of variance on number of trials with the within-subject factors condition (safety, control) and feedback (higher, lower reward). As the original number of trials was 30, we rejected about 10% of trials. According to the literature, 20 artifact-free trials are sufficient to measure a reliable FRN (Marco-Pallares et al. 2011) and a reliable P300 (Cohen and Polich, 1997). Therefore, we consider our mean number of trials as adequate to measure FRN and P300 amplitudes in our study.

Following the recommendation of Sambrook and Goslin (2015), we evaluated the FRN during the period from 270 to 300 ms after monetary reward feedback. FRN amplitude, averaged within this time window, was assessed at electrode FCz, where it reached maximum amplitude in agreement with the literature (Miltner et al. 1997; Sambrook and Goslin 2015). We quantified P300 amplitudes between 328 ms and 380 ms at electrode Pz (Begleiter et al. 1983; Polich 2007). In his review, Polich (2007) defines P300 peak latency as “largest positive-going peak of the ERP waveform within a time window of 250-500 ms”. Concerning the chosen electrode Pz, Polich states “P300 scalp distribution is defined as the amplitude change over the midline electrodes (Fz, Cz, Pz), which typically increases in magnitude from the frontal to parietal electrode sites.” To provide a better overview of our EEG data, we added a Supplementary Figure with the ERPs to reward feedback in both conditions for all electrodes. As described in Polich (2007), the P300 is visible in midline electrodes, increasing from frontal to parietal electrode sites. The maximal amplitudes for P300 appeared at Pz.

## Results

### Safety Ratings and Hypnotic Depth

All participants passed the hypnosis test item in the safety condition after the induction of hypnosis, which is the first item of the Stanford Hypnotic Susceptibility Scale, Form C (Weitzenhoffer and Hilgard 1962). Their arm moved downwards more than 15 cm after the suggestion of carrying a heavy weight in their outstretched right hand. After completing the safety condition, participants indicated that they felt significantly safer, *t*(23) = 25.0, *p* < 0.001, *d* = 5.1. Their mean score on the scale from 1 for “not at all” to 5 for “I felt very safe” was 4.1 (*SD* = 0.6). On the ISHD-D, which measures hypnotic depth, participants scored on average 99.3 (*SD* = 12.4). According to Riegel et al. (2018), ISHD-D scores of at least 95 indicate deep trance. The higher the HGSHS:A score that indicates how suggestible the participant is, the higher was the ISHD-D score indicating the hypnotic depth that the participant achieved during the safety condition, *r* = 0.42, *p* = 0.04. Further, participants spontaneously reported that monetary rewards were less important when they felt safe.

### Risk Game: Behavior

Participants chose one of the presented risk options after on average 1.8 seconds (*SD* = 0.4 seconds). Response times did not differ significantly between conditions (*t*(23) = 0.02, *p* = 1). To get an indicator of risk behavior, we computed the percentage of risky decisions for every participant by dividing the number of trials in the risk game where the participant chose the riskier option by the number of all trials. Participants chose the riskier option on average in 45% of trials (*SD* = 21%). We performed an analysis of variance on percent riskier decisions including the within-subject factors condition (safety, control) and alternative option (10 or 1 cent, 9 or 2 cents, 8 or 3 cents, 7 or 4 cents, 6 or 5 cents). Participants’ risk behavior did not differ significantly between the safety and control condition (*F*(1,23) = 0.2, *p* = 0.6, *η_G_^2^* = 0.002). The riskier the alternative option, the more often participants chose the riskier option, indicated by a significant main effect of alternative option: *F*(4,92) = 4.4, *p* = 0.002, *η_G_^2^* = 0.05. The interaction effect of condition and alternative option did not reach significance (*F*(4,92) = 0.2, *p* = 0.9, *η_G_^2^* = 0.001).

### Risk Game: Option Ratings

**Figure 3:**
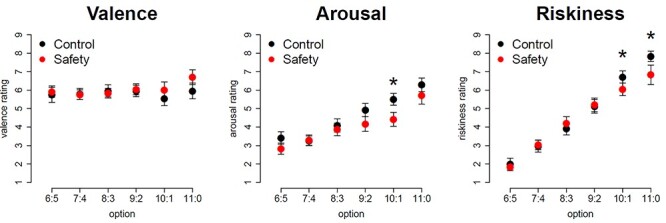
Valence, arousal, and riskiness ratings of all risk options for both the safety and the control condition. Asterisks indicate significant condition differences. Error bars are standard errors of the mean.

We performed separate analyses of variance on the valence, arousal, and riskiness ratings of the risk options, with condition (safety, control) and risk option (6 or 5 cents, 7 or 4 cents, 8 or 3 cents, 9 or 2 cents, 10 or 1 cents, 11 or 0 cents) as within-subject factors. These analyses revealed significant main effects of risk option for arousal and riskiness ratings but not for valence ratings (arousal: *F*(5,115) = 36.1, *p* < 0.001, *η_G_^2^* = 0.25; riskiness: F(5,115) = 104.8, p < 0.001, *η_G_^2^* = 0.56). Visual inspection of the data indicates higher arousal and higher perceived riskiness with increasing objective riskiness of the options (Fig. 3). For riskiness ratings, also the interaction effect of condition and option was significant *F*(5,115) = 5.2, *p* < 0.001, *η_G_^2^* = 0.02. We performed post-hoc *t*-tests to detect significant differences between the safety and control conditions concerning each risk option for valence, arousal and riskiness ratings. Participants rated the second riskiest option 10 or 1 cents as less arousing in the safety condition compared to the control condition *t*(23) = 2.2, *p* = 0.04, *d* = 0.4, indicated by an asterisk in Figure 3. Concerning riskiness ratings, participants rated the second riskiest option 10 or 1 cents and the riskiest option 11 or 0 cents as less risky in the safety condition compared to the control condition, indicated by asterisks in Figure 3 (10 or 1 cents: *t*(23) = 2.1, *p* = 0.04, *d* = 0.4; 11 or 0 cents: *t*(23) = 2.6, *p* = 0.02, *d* = 0.5).

### FRN

**Figure 4:**
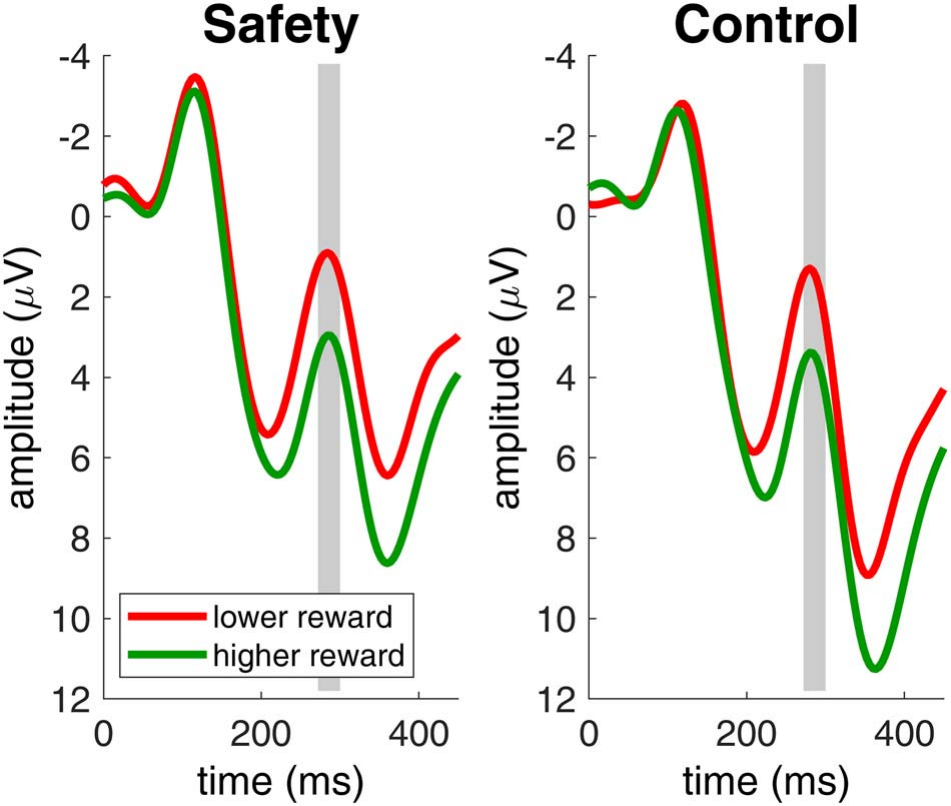
Brain responses to lower and higher monetary rewards in the risk game. Lower monetary rewards elicited more negative FRN amplitudes. The gray area indicates the FRN time window. Data were recorded at channel FCz. Negative is plotted up by convention.

We analyzed the amplitude of the FRN to monetary rewards with an analysis of variance with the within-subject factors condition (safety, control) and reward (lower, higher). After lower monetary rewards, FRN amplitudes were more negative than after higher monetary rewards (*F*(1,23) = 18.8, *p* < 0.001, *η_G_^2^* = 0.03). All other effects did not reach significance (main effect of condition: *F*(1,23) = 0.5, *p* = 0.5, *η_G_^2^* = 0.002; interaction effect of condition and reward: *F*(1,23) = 0.003, *p* = 1, *η_G_^2^* = 0.000004). Figure 4 shows the ERP at electrode FCz with marked FRN time windows for both conditions.

### P300

**Figure 5:**
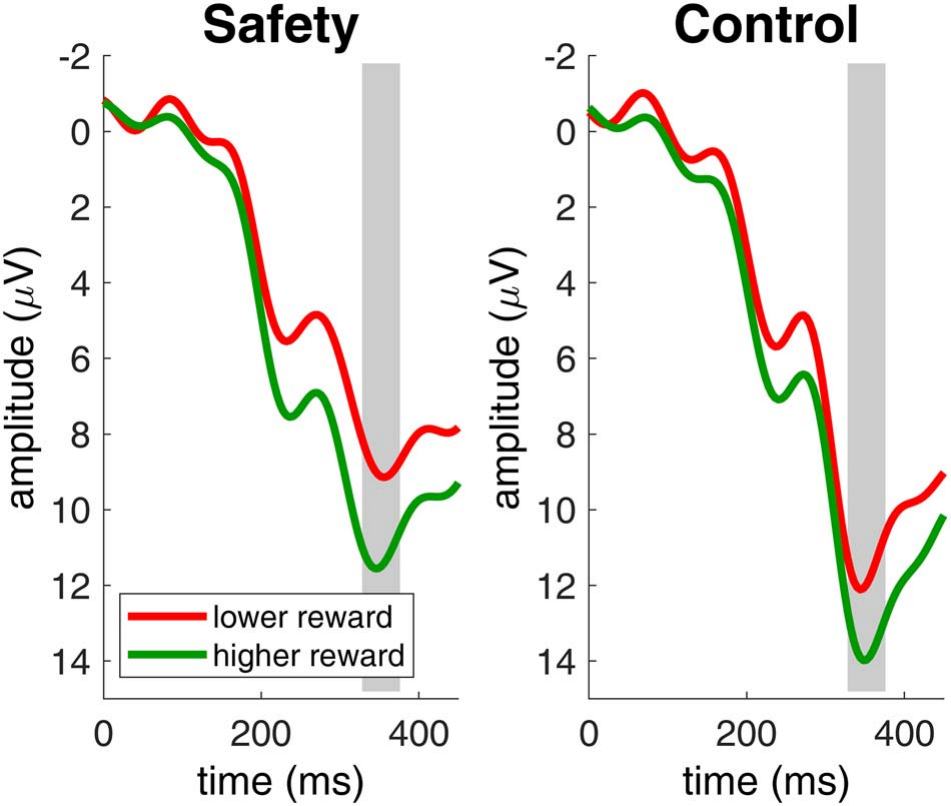
Brain responses to lower and higher monetary rewards in the risk game. Higher monetary rewards elicited higher P300 amplitudes. Participants showed significantly smaller P300 amplitudes to all monetary rewards in the safety condition compared to the control condition. The gray area indicates the P300 time window. Data were recorded at channel Pz. Negative is plotted up by convention.

**Figure 6:**
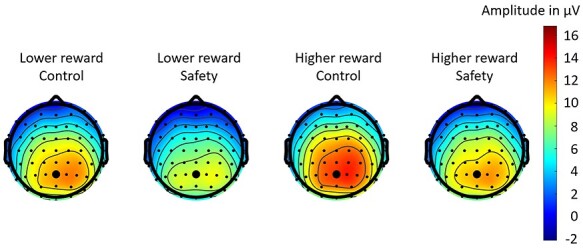
Topographical maps of brain responses in the P300 time window to lower and higher monetary rewards in the risk game. Lower rewards elicited smaller P300 amplitudes than higher rewards. Participants showed significantly smaller P300 amplitudes in the safety condition compared to the control condition. The P300 is maximal over channel Pz, marked by a bigger black dot.

The remainder of our analysis focuses on P300 amplitudes following monetary rewards in the risk game. Figure 5 shows the ERP response to monetary rewards in both conditions at Pz with the P300 time window marked in gray. We performed an analysis of variance on P300 amplitudes with the within-subject factors condition (safety, control) and reward (lower, higher). P300 amplitudes to monetary rewards were significantly reduced in the safety condition compared to the control condition (*F*(1,23) = 8.3, *p* = 0.009, *η_G_^2^* = 0.07). After higher monetary rewards, P300 amplitudes were higher than after lower monetary rewards (*F*(1,23) = 17.7, *p* < 0.001, *η_G_^2^* = 0.05). The interaction effect of condition and reward did not reach significance (*F*(1,23) = 0.2, *p* = 0.6, *η_G_^2^* = 0.0005). In Figure 6, we show the topographical distribution of the P300 effect in the P300 time window (gray shaded area in Fig. 5). As expected, P300 effects were maximal over Pz and showed significantly higher positive amplitudes for higher monetary rewards compared to lower monetary rewards. Most important, P300 amplitudes were significantly reduced in the safety condition compared to the control condition.

## Discussion

In this study, we show that P300 amplitudes to monetary rewards are significantly reduced when participants were suggested to feel safe during hypnosis compared to a control condition. This reduction in P300 amplitudes was in line with participants’ statements that money was less important when they felt safe during hypnosis. This reduction of P300 amplitudes is associated with reduced motivation and attention towards monetary rewards and indicates reduced subjective importance of monetary rewards.

Our results are also in line with neuronal dissociation theories of hypnosis (e.g., Kirsch and Lynn 1998). While earlier processing stages were not affected by the safety suggestion, indicated by non-significant FRN amplitude differences between conditions, later processing stages were affected by the safety suggestion, indicated by the significant reduction of P300 amplitudes in the safety condition. Participants still differentiated between lower and higher rewards, reflected by the significant main effect of reward magnitude for FRN and P300 amplitudes. Also, participants did not show significant differences in their risk behavior between conditions. We conclude that participants correctly detected reward magnitudes in both conditions and played the risk game similarly in both conditions, but cared less about monetary rewards in general in the safety condition.

Importantly, we can rule out the alternative explanation that participants were just so relaxed they did not care about anything during hypnosis. First, response times did not differ significantly between conditions, so participants did not respond significantly slower during hypnosis. Second, arousal ratings of risk options did not differ significantly between conditions, so participants did not rate all options as significantly less arousing during hypnosis. Third, participants perceived differences between positive and negative outcomes in both conditions, indicated by the significant main effect of reward magnitude and a non-significant interaction effect of condition and reward magnitude both for FRN amplitudes and P300 amplitudes. We conclude that the observed effects are not primarily due to generally reduced arousal during hypnosis.

Participants indicated feeling significantly safer during the safety condition, which is a positive feeling. The induction of a positive feeling could lead to a reduction of attention and motivation towards stimuli that normally attract attention and motivation such as monetary rewards. Especially in substance use disorders, it has been shown that craving for substance-related stimuli is explained by escaping from negative affect or by striving for positive affect (Cepeda-Benito et al. 2000; Schlauch et al. 2013). Therefore, being in a positive affective state like feeling safe might reduce the urge to approach substance-related stimuli.

Another important aspect in this regard is the fact that feeling safe did not significantly affect risk behavior of participants. When we consider the possible use of the safety suggestion in substance use disorders, this is an advantage. Riskier behavior is associated with the consumption of drugs (Horvath and Zuckerman 1993) which would be a clear contraindication for participants with substance use disorder. Our results indicate that we do not necessarily expect the negative side effect of riskier behavior after the induction of feeling safe.

Instead of substance-related stimuli, we used monetary rewards in our study. Money is a reinforcing stimulus that increases motivation and attention which has been shown both in field studies and inside the laboratory (Jenkins et al. 1998; Small et al. 2005). Especially in the risk game we used in our study, it has been shown that higher monetary rewards elicit higher P300 amplitudes (Schmidt, Kessler, Hecht et al. 2019). Therefore, a reduction of P300 amplitudes to monetary rewards in the risk game indicates reduced motivation and attention towards those stimuli.

Similar decreases in P300 amplitudes take place when individuals with substance use disorder successfully abstain from their desired substance. Smokers who did not smoke for a mean duration of 1.4 years showed significantly reduced P300 amplitudes to smoking cues compared to active smokers (Littel and Franken 2007). In addition, there were no P300 differences between ex-smokers and non-smokers anymore, providing further evidence that a reduced P300 amplitude to smoking cues is an indicator of reduced attention and motivation towards the addictive substance (Littel and Franken 2007). Please note that these results are specific for P300 amplitudes and do not occur for later components of the ERP like the LPP (Deweese et al. 2018).

Our results are of great relevance for clinical application. When substance-related stimuli lose their subjective importance, P300 amplitudes decrease. This can be a result of a changed state like the state of hypnosis with suggested safety in our study. When this procedure is repeated, it is possible that this changed state turns into a changed trait. That would result in reduced P300 responses as it has been shown in abstinent smokers (Littel and Franken 2007). Encouraging evidence in studies about smoking cessation via hypnosis suggest that hypnosis is a possible treatment for substance-use disorders (Carmody et al. 2009; Hasan et al. 2014). Reducing P300 amplitudes might be a neuronal mechanism for the reduction of motivated attention or subjective importance of substance-related stimuli using hypnosis. Our study is a first step encouraging further research to develop a clinical intervention using the suggestion of safety for substance use disorders.

## Supplementary Material

Supplementary_Figure_tgaa050

Safety_Suggestion_English_tgaa050

Safety_Suggestion_English_tgaa050

Safety_Suggestion_German_tgaa050

Safety_Suggestion_German_tgaa050
