# Supplementary material for: Feel Safe and Money is Less Important! Hypnotic Suggestions of Safety Decrease Brain Responses to Monetary Rewards in a Risk Game
Source: Cereb Cortex Commun. 2020 Aug 20;1(1):tgaa050. doi: 10.1093/texcom/tgaa050 (PMC8152948; doi:10.1093/texcom/tgaa050)
Supplement: Safety_Suggestion_English_tgaa050 [file safety_suggestion_english_tgaa050.docx]

**Suggestion of safety during hypnosis**

Author: Dr. Barbara Schmidt, University of Jena

**Welcome and setting**

I welcome you warmly to this hypnosis session. I will guide you to relax and feel a pleasant feeling of safety. Please take about 30 minutes of your time. I am Dr. Barbara Schmidt and I am currently doing research at the University of Jena on the neuronal basis of hypnosis and decision-making. I have successfully used the text in two studies before, where it showed impressive results. I invite you to sit down in a comfortable chair, place your legs comfortably next to each other and follow my instructions. The state of hypnosis is very pleasant and feels like watching a movie and being completely absorbed in it. Everything around you becomes unimportant and you concentrate on only one thing. Everyone can go into a trance. Some easier than others. Repeated practice will help you to let go and relax. I'm going to teach you how to do it. You'll be in complete control of everything that happens at all times. I just invite you to follow what I am offering you here.

**Hypnosis induction**

Then we start with our hypnosis session. I want you to relax as much as possible while you sit comfortably in your chair.

Just pay attention to what happens and try to allow the experiences I will suggest to you. Do not push yourself to do anything that does not work for you but try not to hold back things that do work. You can do absolutely nothing wrong. Just be completely relaxed.

Pick a point that you can fix during the next few minutes. The point is a little above your head height in a few meters distance. Maybe you have a picture on the wall or a bookshelf? I ask you to relax in your chair, look at your point and listen to my voice. Meanwhile, I will give you some instructions that will help you to relax and gradually enter a hypnotic state.

Please keep your eyes on your point and continue to listen to my words as you look at it. You can only enter a hypnotic state when you want to. Try your best to concentrate on the point and pay attention to my words. If your thoughts wander, just bring your thoughts back to the point and to my words. Pay attention to how the point changes, how it may sometimes become unclear, sometimes clearer. Whatever is going through your mind, allow it, but keep fixing the point.

Relaxation under hypnosis is very similar to the state just before falling asleep, but you will not sleep in the usual sense because you will continue to hear my voice and be able to focus your thoughts on what I am telling you.

You are pleasantly relaxed, but you will relax much more, much more.

Just pay attention to my voice. Sometimes my voice may seem to change or sound as if it is far away. That's fine. If you start to feel sleepier, that's fine too. Accept whatever happens and just keep listening to my voice as you relax more and more.

As you imagine the relaxation, your muscles will relax. Start with your right foot, relax the muscles of your right leg... now the muscles of your left leg... relax completely. Relax your right hand, your forearm, your upper arm and your shoulder... now your left hand... and your forearm... and upper arm... and shoulder... relax your neck and your chest... completely relaxed.

As you relax more and more, your body will feel heavy or maybe numb. You will begin to feel this feeling of numbness or heaviness in your legs and feet... in your hands and arms... in your whole body... as if you are sinking deeper and deeper into the chair. The chair is stable, it will hold your body as it feels heavier and heavier.

Your eyelids feel heavy, very heavy. You begin to feel relaxed and sleepy. Your eyes burn a little bit and your eyelids feel very heavy. Your eyelids are getting heavy...

Your eyes are getting blurred from the effort. You can hardly see your point; your eyes are so strained. Soon you won't be able to keep your eyes open Your eyelids are heavy. Very heavy. Getting heavier and heavier. They press down, deeper and deeper. There seem to be weights on the eyelids, pushing them deeper and deeper. Your eyes are flickering... flickering... closing, closing.

**Your eyes are now closed. Just keep your eyes closed until I ask you to open them.**

You are relaxed, very relaxed. You can relax even more if you just let yourself go. You can reach a state of even deeper, complete relaxation. You are becoming increasingly sleepy. You feel a pleasant feeling of numbness and heaviness throughout your body. You begin to feel so relaxed, so sleepy. It is easier to turn your thoughts away from other things and focus your attention only on my voice. Soon you will only listen drowsily to my voice as you relax more and more.

Now I want you to pay attention to your breathing... Take a deep breath... deep breath. Then hold it... And then exhale through your mouth. Maybe you can already feel peace and relaxation spreading through your body... Now breathe in and out again calmly and evenly - in and out ... As you breathe in, your abdominal wall raises slightly, and as you exhale it lowers slightly again ... in ... and out ... in... and out. How this inhalation and exhalation is accompanied by peace and relaxation! If you continue to breathe in peace, sooner or later you will reach a very pleasant inner peace without having to do anything for it ... You breathe in and out... in and out. Whenever you breathe in, you are taking in oxygen and energy... And every time you exhale you relax more deeply and can let go... With each breath you can let go more... with every single breath... You exhale and let go of everything that disturbs you... You exhale and inner peace will come more and more - all by itself ... And with every breath the relaxation becomes deeper and deeper ... as deep as it is possible for you now.

You don't need to do anything else but pay attention to your breathing and feel how your breath flows in and out ... in and out... And you can imagine how the air you breathe flows through your nose, through your windpipe, to your lungs and back again...

Some people focus their attention on the muscles of the body and feel how they are relaxed deeper and deeper with each breath as if breathing tells the muscles to relax even further ... You do not need to do anything at all... just feel and remember. Often the relaxation can be felt first in the temporal region. It moves from the forehead to the eyes ... to the root of the nose, over the cheeks and nostrils... over the lips and into the mouth.

And this relaxation also spreads to the neck, because with every breath the muscles there also become more relaxed and looser ... You don't have to do anything. Just feel and notice how the relaxation flows - like a stream - over your shoulders, down your back ... Every time you breathe out you can feel the relaxation - even in the upper arms ... in your forearms ... and finally the fingers join in, the palm of your hand ... up to your fingertips ... And when you exhale, the air flows gently and smoothly back out through your nose ... Your arms can relax - all by themselves ... with every breath you take, peace and relaxation become more and more ... If you pay attention to your nose, you can feel the air flowing in and out through your nose ... And you can feel how gradually the whole head area, the upper part of your body and your arms and hands are very relaxed and calm ... While breathing in you can have the feeling of becoming lighter ... This is perfectly normal ... Perhaps you have already noticed that exhaling takes a little longer than inhaling ... So with every breath you take you sink deeper ... and deeper... and deeper.

You are relaxed, very relaxed. Your whole body feels heavy and relaxed. You feel a pleasantly warm feeling in your whole body as you become more and more sleepy. Drowsy. Very sleepy. Continue to focus your thoughts on what I am saying; listen to my voice. Soon you will think of nothing but my voice and the words I say as you relax more and more. There is nothing to worry about now. Nothing except what my voice tells you seems important, nothing else is important now. Even my voice may sound a little strange, as if it was coming from a dream, as you sink deeper into this numbness, this heaviness of deep relaxation. Relax completely...

I will soon start counting from 1 to 10. As I count, you will notice how you fall deeper and deeper into a restful sleep. But you'll still be able to do all the things I ask you to do without waking up.

1 ... You start to relax even deeper ... 2 ... Deep, deep into a deep healthy sleep ... 3, 4 ... sleeping deeper and deeper ... 5, 6, 7, ... You're sinking into a deep, deep sleep. Please direct your thoughts to my voice and the things I'm saying to you. You will experience many of the things I describe to you ... 8, 9, 10 ... You won't wake up until I ask you to.

**Safety suggestion**

I want to take you on a wonderful trip now. We're going to a place where you'll feel very comfortable and safe. Just let my voice lead you to that place. Look forward to the impressions that are waiting there for you. Maybe you remember a place where you already felt good and safe. Go all the way to this place by feeling deeper and deeper into yourself and bring this place inside you to life. Feel yourself entering this place. What exactly do you feel now, what impressions do you feel in this place of security? You feel how peace and serenity come into you and spread out. Allow it. Let yourself fall completely. Your breaths are calm and support you in letting yourself dive into the good feelings that await you in your safe place. With every inhale you gain strength and with every exhale you can let yourself go even more, into the good feeling of safety in this beautiful place. In this place you have the feeling that you are completely at rest within yourself. Everything is fine as it is. You feel a deep satisfaction. The outside world, which might make you feel uncomfortable on other occasions, stays far behind. You are safely shielded, as if a blanket is wrapped around you, warm and protective. You feel safe and secure in your shell of safety. How good it feels to be so relaxed and calm. Feel inside yourself. At what part of your body do you perceive this feeling of security most strongly? When you have found this place in your body, concentrate on it and increase the feeling. The feeling of security grows, just like a plant grows, which gets a lot of light and water and stands on a nearby ground. The place where you are helps you to make this feeling big. The good feeling of security spreads further into your body and makes sure that you relax completely. You also feel deep trust. Allow yourself to feel completely safe. You can let yourself go completely. Think again at the place in your body where you feel the feeling of security most clearly. From there it radiates out into all parts of your body like sunbeams. And you know how strong the sun can shine. It is a thoroughly pleasant feeling. You are completely filled with it. The feeling gets stronger and stronger. It becomes so big that even outside of your body it can be felt like a safe shell. The feeling of safety is like a blanket in which you feel safe and secure. You are now completely enveloped by the feeling of safety. I will begin to count from 1 to 10 and then you will feel completely safe. With every number, your feeling of security and relaxation doubles and you will move deeper into the trance.

1 ... you feel always, always safer... 2 ... you relax twice as deep and feel even safer 3 ... 4... and you sink again 5... 6... you feel safe now 7... 8... 9... 10...

I now invite you to connect this feeling of safety with a certain trigger, so that you can call up the feeling again and again when you need it. The trigger can be a picture, like the picture of the blanket wrapped around you. It can also be a color that you associate with the feeling of safety or even a melody. Now store your feeling of safety within this trigger. When you press the trigger, you will feel the same feeling as you do now. It will be like the trigger that opens a parachute or like the trigger that opens an umbrella. This is your trigger for your feeling of safety and security. So this wonderful feeling is always available to you when you need it. Just activate the trigger, that is your picture, your color or your melody and you will feel this wonderful feeling again.

**Removing hypnosis**

Now imagine yourself return to the here and now.

As I count backwards from 10 right now, you will slowly come back. When I get to 1, you'll be all there again. You will also feel how your relaxation turns into a pleasant feeling of being rested and you will feel fresh and lively at the end. All right, I'll start counting.

10 ... 9 ... 8 ... 7 … 6 … 5 … 4 … 3 … 2 … 1

You have returned to the here and now. Great. Just relax and listen to me. Enjoy these moments a little bit more. A feeling of freshness in your lungs accompanies your breathing and you feel increasingly relaxed. I would like you to take a few more breaths now, inhale deeply through your nose and exhale audibly through your mouth. Feel how you become more lively and fresh with every breath, how confidence and new energy rise inside you. When you are on the last one of these breaths, you can tense your muscles, stretch your body and you are back to the here and now.

Now give yourself a little time to return into your daily routine. I hope you enjoyed our little journey! All the best for you and goodbye!
