# Supplementary material for: Feel Safe and Money is Less Important! Hypnotic Suggestions of Safety Decrease Brain Responses to Monetary Rewards in a Risk Game
Source: Cereb Cortex Commun. 2020 Aug 20;1(1):tgaa050. doi: 10.1093/texcom/tgaa050 (PMC8152948; doi:10.1093/texcom/tgaa050)
Supplement: Safety_Suggestion_German_tgaa050 [file safety_suggestion_german_tgaa050.docx]

**Suggestion von Sicherheit unter Hypnose**

Autorin: Dr. Barbara Schmidt, Universität Jena

**Begrüßung und Setting**

Ich begrüße Sie herzlich zu dieser Hypnose Sitzung. Ich werde Sie dazu anleiten, sich zu entspannen und ein angenehmes Gefühl der Geborgenheit zu spüren. Nehmen Sie sich dafür etwa 30 Minuten Zeit. Ich bin Dr. Barbara Schmidt und forsche derzeit an der Universität Jena zu den neuronalen Grundlagen von Hypnose und Entscheidungsverhalten. Den Text, den ich hier verwende, habe ich bereits in zwei Studien genutzt und er hat sehr gute Effekte erzielt. Ich lade Sie herzlich ein, sich jetzt in einen gemütlichen Stuhl zu setzen, die Beine bequem nebeneinander aufzustellen und meiner Anleitung zu folgen. Der Zustand der Hypnose ist sehr angenehm und fühlt sich in etwa so an, wie wenn Sie gebannt einen Film ansehen und ganz darin versinken. Alles um Sie herum wird unwichtig und Sie konzentrieren sich nur auf eine Sache. Jeder hat die Fähigkeit, in Trance zu gehen. Bei manchen ist sie stärker ausgeprägt als bei anderen. Wiederholtes Üben hilft, damit Sie sich noch besser fallen lassen können. Ich leite Sie jetzt an, wie das geht. Dabei haben Sie zu jedem Zeitpunkt die volle Kontrolle über alles, was passiert. Ich lade Sie dazu ein, dem zu folgen, was ich Ihnen hier anbiete. Im Folgenden werde ich Sie in der du-Form ansprechen, da dann die Hypnose noch besser funktioniert.

**Hypnose Einleitung**

Dann beginnen wir jetzt mit unserer Hypnose-Sitzung. Ich möchte, dass du dich möglichst optimal entspannst und dass du für die nächste Zeit ganz bequem im Stuhl sitzen kannst.

Achte einfach darauf, was passiert und versuche, die Erfahrungen, die ich dir suggerieren werde, zuzulassen. Dränge dich zu nichts, was nicht funktioniert, aber versuche Dinge, die gelingen, nicht zurückzuhalten. Du kannst absolut nichts falsch machen. Sei einfach ganz entspannt.

Suche dir einen Punkt aus, den du in den nächsten Minuten fixieren kannst. Der Punkt liegt etwas über deiner Kopfhöhe in ein paar Metern Entfernung. Vielleicht hast du ein Bild an der Wand oder ein Bücherregal? Ich bitte dich, dich im Stuhl zu entspannen, deinen Blick auf deinen Punkt zu richten und meiner Stimme zuzuhören. Währenddessen werde ich dir einige Instruktionen geben, die dir dabei helfen werden, dich zu entspannen und nach und nach in einen hypnotischen Zustand zu gelangen.

Bitte lasse deinen Blick unentwegt auf deinem Punkt und achte weiterhin auf meine Worte, während du ihn betrachtest. Du kannst nur dann in einen hypnotischen Zustand gelangen, wenn du selbst es willst. Versuche dein Bestes, dich auf den Punkt zu konzentrieren und aufmerksam auf meine Worte zu achten. Falls deine Gedanken wandern, bringe deine Gedanken einfach zurück auf den Punkt und auf meine Worte. Achte darauf, wie sich der Punkt verändert, wie er vielleicht manchmal unklar, manchmal klarer wird. Was immer dir durch den Kopf geht, lasse es zu, aber fixiere weiterhin den Punkt.

Die Entspannung in Hypnose ähnelt sehr dem Zustand kurz vor dem Einschlafen, aber du wirst nicht im üblichen Sinne schlafen, weil du weiterhin meine Stimme hören wirst und in der Lage sein wirst, deine Gedanken auf das zu richten, was ich dir sage.

Du bist angenehm entspannt, aber du wirst dich noch viel mehr entspannen, viel mehr.

Achte einfach aufmerksam auf meine Stimme. Manchmal scheint sich meine Stimme vielleicht zu ändern oder hört sich vielleicht so an, als wäre sie weit entfernt. Das ist in Ordnung. Wenn du beginnst, dich schläfriger zu fühlen, ist das auch in Ordnung. Akzeptiere, was immer geschieht, und höre einfach weiter meiner Stimme zu, während du dich mehr und mehr entspannst.

Während du dir die Entspannung vorstellst, werden sich deine Muskeln entspannen. Beginne mit dem rechten Fuß, entspanne die Muskeln deines rechten Beines… nun die Muskeln deines linken Beines… entspanne dich vollkommen. Entspanne deine rechte Hand, deinen Unterarm, deinen Oberarm und deine Schulter… nun deine linke Hand… und deinen Unterarm… und Oberarm… und Schulter… entspanne deinen Nacken und deine Brust... vollkommen entspannt.

Während du dich mehr und mehr entspannst, wird sich dein Körper schwer anfühlen oder vielleicht taub. Du wirst beginnen, dieses Gefühl der Taubheit oder Schwere in deinen Beinen und Füßen zu spüren… In deinen Händen und Armen… Im ganzen Körper… So, als ob du zunehmend tiefer in den Stuhl sinkst. Der Stuhl ist stabil, er wird deinen Körper halten, während er sich schwerer und schwerer anfühlt.

Deine Augenlider fühlen sich schwer an, ganz schwer. Du fängst an, dich entspannt und schläfrig zu fühlen. Deine Augen brennen ein wenig und deine Augenlider fühlen sich sehr schwer an. Deine Augenlider werden schwerer...

Deine Augen werden verschwommen von der Anstrengung. Du kannst kaum mehr deinen Punkt sehen, deine Augen sind so angestrengt. Bald wirst du deine Augen nicht mehr offenhalten können. Deine Augenlider sind schwer. Sehr schwer. Werden schwerer und schwerer. Sie drücken nach unten, tiefer und tiefer. Auf den Augenlidern scheinen Gewichte zu liegen, die sie tiefer und tiefer drücken…. Deine Augen flimmern… flimmern … schließen sich, schließen sich.

**Deine Augen sind nun geschlossen.** Lasse deine Augen einfach geschlossen, bis ich dich darum bitte, sie zu öffnen.

Du bist entspannt, sehr entspannt. Du kannst dich noch mehr entspannen, wenn du dich einfach fallen lässt. Du kannst einen Zustand noch tieferer, vollkommener Entspannung erreichen. Du wirst zunehmend schläfrig. Du spürst ein angenehmes Gefühl der Taubheit und Schwere in deinem gesamten Körper. Du fängst an, dich so entspannt zu fühlen, so schläfrig. Es ist einfacher, deine Gedanken von anderen Dingen abzuwenden und deine Aufmerksamkeit allein auf meine Stimme zu richten. Bald wirst du nur schläfrig meiner Stimme zuhören, während du dich mehr und mehr entspannst.

Ich möchte nun, dass du auf deine Atmung achtest ... Atme einmal tief ein…tief ein. Dann halte die Luft an ... Und dann mit einem Mal durch den Mund ausatmen. Vielleicht spürst du schon, wie sich Ruhe und Entspannung in deinem Körper ausbreiten ... Atme nun wieder ruhig und gleichmäßig ein und aus – ein und aus ... Beim Einatmen wölbt sich deine Bauchdecke leicht nach oben, und beim Ausatmen fällt sie wieder leicht nach unten ... ein ... und aus ... ein ... und aus. Wie doch dieses Ein- und Ausatmen mit Ruhe und Entspannung einhergeht! Wenn du in aller Ruhe so weiter atmest, kommst du früher oder später in eine sehr angenehme innere Ruhe, ohne irgendetwas dafür tun zu müssen... Du atmest ein und aus ... ein und aus. Immer, wenn du einatmest, nimmst du Sauerstoff und Energie auf ... Und immer, wenn du ausatmest, entspannst du tiefer und kannst dabei loslassen ... Mit jedem Atemzug kannst du mehr loslassen ... mit jedem einzelnen Atemzug ... Du atmest aus und lässt dabei alles Störende los... Du atmest aus und innere Ruhe wird mehr und mehr – ganz von selbst kommen ... Und mit jedem Atemzug wird auch die Entspannung tiefer und tiefer ... so tief, wie es dir jetzt möglich ist.

Du brauchst nichts anderes zu tun als auf deine Atmung zu achten und zu spüren, wie dein Atem ein- und ausströmt ... ein und aus ... Und du kannst dir vorstellen, wie deine Atemluft durch die Nase über die Luftröhre zur Lunge strömt und wieder zurück...

Manche richten ihre Aufmerksamkeit auf die Muskeln des Körpers und spüren, wie sie mit jedem Atemzug wie von selbst immer tiefer entspannt werden, so, als ob die Atmung den Muskeln mitteilt, sich noch weiter zu entspannen ... Du brauchst dafür überhaupt nichts zu tun ... einfach spüren und merken. Oft kann man die Entspannung als erstes in der Schläfengegend spüren. Sie zieht über die Stirn zu den Augen ... zur Nasenwurzel über die Wangen und die Nasenflügel ... über die Lippen in den Mund hinein.

Und diese Entspannung breitet sich auch aus bis zum Nacken, denn mit jedem Atemzug werden auch dort die Muskeln entspannter und lockerer ... Man muss gar nichts tun. Einfach spüren und merken, wie die Entspannung fortfließt – wie ein Strom – über die Schultern, den Rücken hinab ... Jedes Mal beim Ausatmen kannst du die Entspannung spüren - auch in den Oberarmen ... in deinen Unterarmen ... und schließlich gesellen sich die Finger dazu, die Handinnenfläche ... bis in deine Fingerspitzen hinein ... Und beim Ausatmen fließt die Luft wieder sanft und geschmeidig zurück über die Nase nach draußen ... Dabei können sich deine Arme entspannen - ganz von selbst ... mit jedem Atemzug werden Ruhe und Entspannung mehr und mehr ... Wenn du auf deine Nase achtest, kannst du merken, wie die Atemluft durch deine Nase ein- und ausströmt ... Und du kannst nachfühlen wie allmählich der ganze Kopfbereich, der Oberkörper und deine Arme und Hände von einem tiefen Gefühl der Entspannung und Ruhe ergriffen werden ... Beim Einatmen kann man das Empfinden haben, leichter zu werden ... Das ist ganz normal… Vielleicht ist dir schon aufgefallen, dass das Ausatmen etwas länger dauert als das Einatmen… So sinkst du mit jedem Atemzug tiefer ... und tiefer ... und tiefer.

Du bist entspannt, sehr entspannt. Dein ganzer Körper fühlt sich schwer und entspannt an. Du fühlst ein angenehm warmes Gefühl in deinem ganzen Körper, während du mehr und mehr schläfrig wirst. Schläfrig. Ganz schläfrig. Richte deine Gedanken weiterhin auf das, was ich sage; höre meiner Stimme zu. Bald wirst du an nichts anderes denken als an meine Stimme und an die Worte, die ich sage, während du dich mehr und mehr entspannst. Es gibt nichts, um das du dich jetzt kümmern musst. Nichts außer dem, was meine Stimme dir sagt, scheint wichtig, nichts anderes ist jetzt wichtig. Sogar meine Stimme mag sich etwas fremd anhören, als ob sie einem Traum entspringt, während du tiefer in diese Taubheit sinkst, diese Schwere der tiefen Entspannung. Entspanne dich vollkommen…

Ich werde bald beginnen, von 1 bis 10 zu zählen. Während ich zähle, wirst du bemerken, wie du tiefer und tiefer in einen erholsamen Schlaf fällst. Aber du wirst immer noch in der Lage sein, all die Dinge zu tun, um die ich dich bitte, ohne aufzuwachen…

1… Du beginnst, noch tiefer zu entspannen… 2 …. Tief, tief in einen tiefen gesunden Schlaf … 3, 4… tiefer und tiefer schlafend… 5, 6, 7, …. Du sinkst in einen tiefen, tiefen Schlaf. Nichts wird dich stören… Richte bitte deine Gedanken auf meine Stimme und auf die Dinge, die ich dir sage. Du wirst viele der Dinge erfahren, die ich dir beschreibe,… 8, 9, 10… Tief schlafend. Du wirst nicht aufwachen, bis ich dich darum bitte.

**Sicherheits-Suggestion**

Ich möchte jetzt mit dir eine schöne Reise unternehmen. Und zwar gehen wir zusammen an einen Ort, an dem du dich sehr wohl und sicher fühlst. Lasse dich einfach von meiner Stimme an diesen Ort führen. Freue dich auf die Eindrücke, die dich dort erwarten. Vielleicht erinnerst du dich an einen Ort, an dem du dich bereits gut und sicher gefühlt hast. Gehe den Weg an diesen Ort, indem du immer tiefer und tiefer in dich hineinspürst und diesen Ort in dir zum Leben erweckst. Fühle genau, wie du diesen Ort betrittst. Was genau fühlst du jetzt, welche Eindrücke empfindest du an diesem Ort der Sicherheit? Du spürst, wie Ruhe und Gelassenheit in dir einkehren und sich ausbreiten. Lasse es zu. Lass dich ganz fallen. Deine Atemzüge sind ruhig und unterstützen dich dabei, dich ganz auf die guten Gefühle einzulassen, die dich an deinem sicheren Ort erwarten. Mit jedem Einatmen schöpfst du Kraft und mit jedem Ausatmen kannst du dich noch mehr fallen lassen, hinein in das gute Gefühl der Sicherheit an diesem schönen Ort. An diesem Ort hast du das Gefühl, dass du ganz in dir selbst ruhst. Alles ist in Ordnung, so wie es ist. Du spürst eine tiefe Zufriedenheit. Die Außenwelt, die dich zu anderen Gelegenheiten vielleicht aus der Ruhe bringt, bleibt weit zurück. Du bist sicher abgeschirmt, als ob sich eine Decke warm und schützend um dich legt. Du fühlst dich sicher und geborgen in deiner Hülle aus Sicherheit. Wie gut es sich anfühlt, so entspannt und gelassen zu sein. Spüre in dich hinein. An welcher Stelle deines Körpers nimmst du dieses Gefühl der Sicherheit am stärksten wahr? Wenn du diese Stelle in deinem Körper gefunden hast, konzentriere dich auf die Stelle und vergrößere dadurch das Gefühl. Das Gefühl der Sicherheit wächst, so wie eine Pflanze wächst, die viel Licht und Wasser bekommt und auf einem nahhaften Boden steht. Der Ort, an dem du bist, hilft dir dabei, dieses Gefühl groß werden zu lassen. Das gute Gefühl der Sicherheit breitet sich weiter in deinem Körper aus und sorgt dafür, dass du dich komplett entspannst. Du fühlst auch ein tiefes Vertrauen. Lasse es zu, dass du dich ganz sicher fühlst. Du kannst dich ganz fallen lassen. Denke noch einmal an die Stelle in deinem Körper, wo du das Gefühl der Sicherheit am deutlichsten wahrnimmst. Von dort aus strahlt es aus in alle Teile deines Körpers wie Sonnenstrahlen. Und du weißt wie stark die Sonne strahlen kann. Es ist ein durch und durch angenehmes Gefühl. Du bist ganz erfüllt davon. Das Gefühl wird stärker und stärker. Es wird so groß, dass es sogar außerhalb deines Körpers zu spüren ist wie eine sichere Hülle. Das Gefühl der Sicherheit ist wie eine Decke, in der du dich sicher und geborgen fühlst. Du bist nun ganz von dem Gefühl der Sicherheit eingehüllt. Ich werde gleich beginnen, von 1 bis 10 zu zählen und dann fühlst du dich vollkommen sicher. Mit jeder Zahl, die ich nenne, verdoppelt sich dein Gefühl der Sicherheit und Entspannung und du versetzt dich tiefer in die Trance.

1 ... immer, immer sicherer fühlst du dich… 2... du sinkst in doppelt so tiefe Entspannung und fühlst dich noch sicherer 3... 4 ...und du sinkst noch einmal so tief 5... 6... du fühlst dich jetzt ganz sicher 7... 8... 9... 10...

Ich lade dich jetzt ein, dieses Gefühl der Geborgenheit mit einem bestimmten Auslöser zu verbinden, so dass du das Gefühl immer wieder aufrufen kannst, wenn du es brauchst. Der Auslöser kann ein Bild sein, wie das Bild der Decke, die dich einhüllt. Es kann auch eine Farbe sein, die du mit dem Gefühl der Geborgenheit verbindest oder eine Melodie. Speichere jetzt dein Sicherheitsgefühl in diesem Auslöser. Wenn du den Auslöser betätigst, wirst du das Gefühl wieder genauso spüren wie jetzt. Es wird sein wie der Auslöser, der einen Fallschirm öffnet oder wie der Auslöser, der einen Schirm aufspannt. Das ist dein Auslöser für dein Gefühl der Sicherheit und Geborgenheit. Damit steht dir dieses wunderbare Gefühl immer zur Verfügung, wenn du es brauchst. Aktiviere einfach den Auslöser, also dein Bild, deine Farbe oder deine Melodie und du wirst dieses Gefühl wieder spüren.

**Aufheben der Hypnose**

Stelle dir nun vor, wie du ins Hier und Jetzt zurückkehrst.

Wenn ich jetzt gleich von 10 ab rückwärts zähle, kommst du langsam zurück. Wenn ich bei 1 angekommen bin, bist du wieder ganz da. Dabei wirst du auch spüren, wie deine Entspannung in ein angenehmes Gefühl des Ausgeruht Seins übergeht und du dich am Ende ganz frisch und munter fühlst. Gut, ich fange an zu zählen.

10 ... 9 ... 8 ... 7 … 6 … 5 … 4 … 3 … 2 … 1

Du bist wieder ins Hier und Jetzt zurückgekehrt. Prima. Bleibe noch entspannt und höre mir zu. Genieße diese Augenblicke noch ein bisschen. Ein Gefühl der Frische in den Lungen begleitet deine Atmung und du fühlst dich zunehmend gelöst. Ich möchte, dass du jetzt ganz bewusst noch ein paar Atemzüge nimmst und tief durch die Nase einatmest und hörbar durch den Mund ausatmest. Spüre dabei, wie du mit jedem Atemzug munterer und frischer wirst, wie Zuversicht und neue Energie in dir aufsteigen. Wenn du beim vorletzten dieser Atemzüge bist, kannst du deine Muskeln anspannen und dich räkeln und bist wieder ganz im Hier und Jetzt zurück.

Gib dir jetzt ein bisschen Zeit, um wieder anzukommen. Ich hoffe, du konntest die kleine Reise genießen! Alles Gute und auf Wiedersehen!
